# Governing waste and producing environmental subjects: a sociological analysis of decentralised solid waste management model in Kerala, India

**DOI:** 10.3389/fsoc.2026.1840755

**Published:** 2026-07-24

**Authors:** A. S. Arya, Rahul Shukla

**Affiliations:** School of Social Sciences and Languages, Vellore Institute of Technology, Vellore, Tamil Nadu, India

**Keywords:** decentralized waste management, governmentality, Haritha Karma Sena, social capital, sustainable development, women empowerment

## Abstract

This study examines decentralized solid waste governance in Kerala through the institutional framework of the Haritha Karma Sena (HKS). While existing research on waste management primarily focuses on technical and policy implementation aspects, limited attention has been given to its sociological dimensions. Using qualitative document analysis of policy documents, institutional reports, and operational guidelines, the study analyzes how decentralized governance is structured and represented within official discourse. Drawing on governmentality, structuration theory, and social capital, the paper interprets how policy frameworks are designed to promote environmental responsibility, institutional participation, and social inclusion. The analysis suggests that the HKS model constructs conditions that may enable shifts in social relations, visibility of women workers, and the potential for community engagement. However, the study does not empirically verify these outcomes and highlights the need for future field-based research to examine lived experiences and implementation dynamics.

## 1 Introduction

Municipal solid waste management has become one of the most pressing challenges of environmental governance, as rapid urbanization, population growth, and changing consumption patterns continue to expand both the volume and complexity of waste generation, particularly in developing countries (Wilson et al., 2006; Guerrero et al., 2013). Effective waste governance is now understood to depend less on technical fixes for collection, transportation, and disposal than on institutional coordination, regulatory design, and the active participation of governments, local authorities, communities, and households (Wilson et al., 2006; Guerrero et al., 2013).

In India, this governance challenge has been shaped by a layered legal and constitutional architecture. The Environment (Protection) Act of 1986 first empowered the central government to act on environmental protection, and judicial intervention by the Supreme Court in 1999 led to the Municipal Solid Waste (Management and Handling) Rules, 2000, which assigned local authorities’ primary responsibility for waste collection and disposal. The Solid Waste Management Rules, 2016 extended this framework considerably, mandating source segregation and door-to-door collection while extending responsibility to waste generators themselves (Government of India, 2016). These statutory developments rest on constitutional foundations: Article 48A directs the state to protect the environment, Article 51A(g) makes environmental protection a citizen’s duty, and Articles 243G and 243W—introduced by the 73rd and 74th Constitutional Amendments—devolve planning and implementation authority to Panchayati Raj Institutions and Urban Local Bodies (Agrawal, 1999). Together, these provisions position decentralized, citizen-engaged waste governance as both a constitutional mandate and an administrative reality.

Kerala has emerged as a distinctive example of this decentralized model through the Haritha Karma Sena (HKS). Constituted under the Local Self-Government Department and recruited through the Kudumbashree Mission, HKS is a women-led, community-based system responsible for door-to-door collection of segregated waste across the state’s local self-government institutions (Local Self Government Department, Government of Kerala, 2021). Operating at the interface between households and local government, HKS translates policy mandates into everyday waste-management practice while simultaneously functioning as a livelihood programme for women from economically marginalized backgrounds.

The significance of HKS extends well beyond its environmental function. By positioning women’s collectives as frontline governance actors, the programme intersects simultaneously with questions of livelihood, local governance, community participation, and the social organization of labour historically structured by caste and gender. This makes HKS a particularly instructive case for understanding how environmental governance operates through community-based institutions, and how state interventions become embedded in everyday social relations—a question of particular salience in the Global South, where environmental governance frequently intersects with deeper structures of inequality and participation (Schlosberg, 2007; Nightingale, 2017). Against this backdrop, the present study examines the Haritha Karma Sena as a model of decentralized environmental governance and explores its broader sociological implications.

It is guided by three interrelated research questions:

- How does the institutional architecture and operational practices of HKS reconfigure waste governance? This question examines the multilevel governance structure, technological interventions, and the routinized procedures that outline the operational landscape of HKS.
- How might HKS’s institutional design create conditions for unsettling the caste and gender hierarchies that have conventionally underpinned labour in waste? This question foregrounds the social transformations that the semi-formalization and professionalization of waste work under state auspices may enable.
- How does HKS theoretically serve as a site of governmentality, environmentality, structuration, and social capital formation? This question evaluates the theoretical positions underlying the study of HKS as a complex governance assemblage with relevance beyond the management of waste.

## 2 Literature review

Scholarship on municipal solid waste management has progressively moved from a narrowly technical framing—centred on collection, transport, and disposal—toward an understanding of waste management as a governance problem, shaped by institutional design, policy frameworks, and the distribution of participation across stakeholders (Wilson et al., 2006; Guerrero et al., 2013). This shift has opened space for more critical accounts of whose knowledge and labour governance systems recognize. Rajendra and Sarin (2023), for instance, show that governance frameworks often privilege visible metrics of cleanliness while erasing the embodied experience of waste workers, reproducing caste and gender hierarchies in the process. Waste governance, on this account, is not simply a question of institutional efficiency but of whose contributions are made visible within official discourse.

Decentralization has long been promoted as a strategy for improving accountability and participation in environmental governance, but the evidence on whether it delivers these benefits is mixed. Agrawal (1999) argue that the benefits of decentralization materialize only when downward accountability, not merely the formal transfer of administrative power is built into local institutions; without it, decentralization risks becoming a procedural exercise rather than a substantive shift in who governs. India’s own decentralization framework, anchored in the 73rd and 74th Constitutional Amendments and operationalized through the Government of India (2000) and Solid Waste Management Rules (2016), assigns substantial responsibility to local bodies (Government of India, 2016), yet Rajendra and Sarin (2023) show that flagship instruments such as the Swachh Survekshan cleanliness survey can valorize surface-level indicators while sidelining the realities of marginalized workers. Comparable evidence from Pune indicates that decentralized systems achieve genuine efficiency only where informal waste pickers are actively integrated into governance structures rather than displaced by them (Chikarmane and Narayan, 2005). Decentralization, in other words, must be evaluated not only on administrative grounds but on its capacity to disrupt or simply relocate existing inequalities.

The relationship between waste work and social inequality is well documented in environmental justice scholarship. Waste-related occupations in India have historically been structured along caste lines, with Dalit communities disproportionately concentrated in hazardous, stigmatized roles (Gill, 2009; Gidwani and Reddy, 2011). Pastor et al. (2024) find that mistrust, caste-based stigma, and municipal inaction combine to obstruct the integration of informal waste workers into formal systems, even where policy explicitly calls for such integration. Gender compounds these dynamics: Rajendra and Sarin (2023) show that women’s contributions to waste work are routinely erased from official narratives, reinforcing both occupational stigma and the limits on their recognition, while Harriss-White (2020) demonstrates that India’s waste economy exemplifies the durability of stigmatized labour regimes in which caste and gender jointly reproduce exclusion.

This body of work establishes waste governance as a site where institutional design, decentralization, and entrenched social hierarchy intersect but it engages this intersection largely through the lens of policy outcomes and labour conditions, rather than through the theoretical traditions that explain how governance reaches into everyday conduct and social relations in the first place. Three such traditions are relevant here. Foucault’s concept of governmentality directs attention to the diffuse, productive forms of power through which institutions shape conduct without resorting to direct coercion (Foucault, 1991); Agrawal’s (2005) related notion of environmentality extends this to the environmental domain, examining how individuals come to take up responsibility for nature as part of their own self-understanding. Within governmentality studies, scholars have emphasized the role of technical instruments audits, standards, monitoring in operationalizing governance at a distance (Rose and Miller, 2013; Rose, 1999), while others caution that governmentality analysis must move beyond description to address the politics of inequality, agency, and contestation embedded in such arrangements (O'Malley et al., 1997). Giddens’s (1984) structuration theory offers a complementary lens, conceptualizing social structures as simultaneously the medium and outcome of practice, useful for examining how the routinized, repeated interactions of frontline workers might reshape institutional norms over time. Finally, social capital theory (Putnam et al., 1994; Granovetter, 1973) provides a vocabulary for the networks of trust and reciprocity that decentralized governance depends upon, with Evans (1996) and Uphoff (2000) showing that such capital is not simply a precondition for state action but can itself be generated and strengthened through state-led programmes.

Despite their evident relevance to cases like HKS, these three traditions have rarely been brought into conversation with one another, and even more rarely applied to the specific context of decentralized, women-led waste governance in India. Existing scholarship has examined institutional design, decentralization’s limits, and caste-gender exclusion in waste work largely as separate lines of inquiry; what remains absent is an integrative analysis of how governmentality, structuration, and social capital operate together within a single governance assemblage. This gap is the starting point for the present study, whose guiding research questions are outlined in the Introduction above.

The study makes three key contributions to the literature. First, it presents a detailed institutional analysis of Kerala’s decentralized waste management regime, elaborating upon the multilevel governance architecture, operational mechanisms, training systems, waste processing infrastructure, and digital platforms that comprise HKS. This systematic documentation addresses an empirical lacuna in the literature on large-scale, state-led waste management reforms in the Global South. Second, the paper situates HKS within broader debates on governmentality and the constitution of environmental subjectivity, showing how everyday waste management practices serve as technologies of environmental governance. Third, it analyzes HKS’s disruptive potential for entrenched social hierarchies, contributing to literature on environmental justice and the political economy of waste in caste-stratified societies.

## 3 Methodology

This study adopts a qualitative policy and practice review of Kerala’s decentralized solid waste management system, based on a document-based case analysis of official policy and institutional texts. It employs structured qualitative document analysis (QDA) alongside sociological interpretation through the analytical frameworks of governmentality, structuration, and social capital. Positioned at the intersection of policy analysis and sociological inquiry, the study treats QDA as a systematic and rigorous methodological approach for examining policy texts, institutional documents, and governance frameworks, consistent with established methodological standards in qualitative research (Altheide et al., 2008; Bowen, 2009).

### 3.1 Data sources and selection criteria

Document selection was guided by the following criteria:

- *Relevance:* inclusion of documents directly related to decentralized solid waste management and the operationalization of the Haritha Karma Sena (HKS)
- *Institutional authority:* preference for materials produced by government departments, state missions, and officially recognized agencies
- *Analytical depth:* selection of documents containing detailed accounts of governance structures, operational mechanisms, training systems, and policy objectives

The dataset includes the following categories:

- *Policy documents*: government orders, operational guidelines, and policy frameworks issued by Kerala’s Local Self-Government Department, including the Government of Kerala (2018) and SWM Rules implementation directives
- *Institutional reports*: annual reports and operational documentation from the Haritha Kerala Mission, Clean Kerala Company Limited, Suchitwa Mission, and Kudumbashree State Mission
- *Legislative and regulatory texts*: constitutional provisions, the Government of India (1986), Government of India (2016), and related regulatory frameworks
- *Technical documentation*: operational manuals, training modules from EKSAT (Empowerment through Knowledge, Skill, Attitudinal Change and Traits), and guidelines for Material Collection Facilities (MCFs) and Resource Recovery Facilities (RRFs)
- *Digital governance documentation*: technical specifications and user guidelines for the Haritha Mitram application developed by KELTRON

These documents were treated not merely as descriptive sources but as institutional texts that encode governance rationalities, regulatory frameworks, and normative expectations, enabling a theoretically informed interpretation of decentralized environmental governance.

### 3.2 Analytical framework

#### 3.2.1 Stage 1: systematic reading and data organization

The study systematically identified the principal institutional actors, governance mechanisms, operational procedures, and articulated policy objectives. This phase thus emphasized mapping the formal architecture of HKS and its integration in Kerala’s wider decentralized governance framework.

#### 3.2.2 Stage 2: thematic coding

Using Braun and Clarke’s (2006) thematic analysis framework, the research identified salient patterns within the documents, namely: (a) institutional roles and networks; (b) professional transition of waste workers; (c) the use of technology for governance and oversight; (d) community engagement strategies; (e) instruments promoting gender and social inclusion; and (f) linking up with sustainability objectives.

#### 3.2.3 Stage: 3 theoretical interpretation

The coded themes were subsequently interpreted through three theoretical lenses namely, governmentality studies, structuration theory and social capital theory to generate analytical insights extending beyond descriptive accounts. This interpretive stage attempted to theorize HKS as a governance assemblage with implications for understanding environmental subjectivity formation, structural transformation through practice, and the role of social networks in environmental compliance.

## 4 Analysis

### 4.1 Kerala’s decentralized solid waste management framework

Prior to the 2016 Solid Waste Management Rules of India, Kerala followed a decentralized approach in solid waste management, which differs from the centralized model. This kind of shift exemplifies the unique political culture of the state and promotion of subsidiarity within participatory governance (Heller and Thomas Isaac, 2003; Heller, 2004). Kerala’s territorial composition includes 941 Grama Panchayats, 87 Municipalities, and 6 Corporations. The generation of solid waste per day is about 11,449 tons, out of which 3,452 tons per day (TPD) are urban and 7,997 TPD are rural (Government of Kerala, 2022).

Decentralization involves the management of waste right at the source, that is, households, institutions, or community sites, which, in turn, allows for the avoidance of long-distance transportation to central facilities. According to Ganesan (2017), this model requires a set of changes in behavior at the household level and coordination in the levels of governance. This was articulated in the Government of Kerala (2018) and also accords with Rules 11 and 15 of the India’s SWM Rules, 2016. It pursues two parallel modes: (a) DSWM, which involves segregation at source and community levels and processing, and (b) centralized operations for cases where decentralization is not feasible due to technical or economic reasons. This two-track system will meet Kerala’s varied waste generation streams, infrastructural capabilities, and geography.

### 4.2 Policy objectives and strategic pillars

The Solid Waste Management Policy of Kerala articulates eleven interrelated objectives designed to transform the state into “a garbage-free state and thereby an environmentally healthy state” (Government of Kerala, 2018, p. 9). These objectives include: raising public awareness through campaigns to “generate awareness about the responsibility of citizen, institutions and community to manage the waste generated by them” (p. 9); promoting behavioral transformation by “bring[ing] about and sustain(ing) behavioral change to segregate waste at source based on its characteristics” (p. 9); strengthening health-environment linkages by “creat(ing) awareness about the linkage of waste management with public health and environmental cleanliness” (p. 9); encouraging adoption of advanced technologies for processing biodegradable and non-biodegradable waste; ensuring accessibility and inclusion by providing community-level facilities for households lacking resources; maximizing resource efficiency through reduction, reuse, and recycling; supporting entrepreneurship in waste-based initiatives; mainstreaming waste management across governance sectors; empowering institutions by “strengthen(ing) the urban and rural local governments as well as public and private institutions and community to accord priority to waste management actions” (p. 9); safeguarding workers by “ensur(ing) environmental, social and safety linked safeguards for those involved in waste handling” (p. 9); and developing human resources through systematic training and deployment. Together, these objectives provide a comprehensive framework that integrates citizen responsibility, institutional capacity, technological innovation, and worker protection into Kerala’s vision of decentralized waste governance.

### 4.3 Strategic implementation mechanisms

The policy operationalizes these objectives through a comprehensive strategic framework emphasizing:

- Source segregation mandate: compulsory separation of waste at household level into biodegradable, non-biodegradable, and hazardous categories.
- Composting infrastructure: promotion of both aerobic and anaerobic composting technologies at household and community levels, including aerobins, pipe compost systems, compost pits, kitchen bins, and biogas plants.
- Collection systems: door-to-door collection and bulk handling protocols for non-biodegradable waste, alongside specialized procedures for domestic hazardous waste.
- Public infrastructure: deployment of segregated waste storage bins in commercial and institutional settings; mandated in-house waste management systems for bulk generators.
- Advanced processing technologies: establishment of centralized waste processing facilities in urban centers and development of regional sanitary landfills for non-recyclable residues.
- Circular economy integration: leveraging processed waste (particularly compost) for organic agriculture and ecosystem conservation through the Green Kerala Mission.
- Regulatory mechanisms: discouragement of single-use items, rationalized penalty structures, and strengthened enforcement protocols.
- Digital governance: automated grievance redressal systems and digital monitoring platforms.
- Capacity building: systematic stakeholder training, public awareness campaigns, and community monitoring systems.
- Innovation ecosystems: collaboration with academic and R&D (Research and Development) institutions for technology development; promotion of waste sector startups targeting skilled youth and professionals (Government of Kerala, 2018).

### 4.4 Institutional architecture

The decentralized solid waste management system in Kerala works through a multilevel institutional framework where each stakeholder has distinct yet interrelated roles and responsibilities. At the top is the local self-government institution, which has service delivery and regulatory enforcement responsibilities. It possesses constitutional authority (Articles 243G and 243 W) to implement waste management plans, levy user fees, and enforce compliance and Suchitwa Mission functions as a technical backstopping agency, providing financial support, technical guidance, and capacity-building services to LSGIs for effective waste management service provision. Along with clean Kerala company limited (CKCL), a state-owned enterprise mandated commercial handling of nonbiodegradable waste, establishing material collection facilities (MCFs) and resource recovery facilities (RRFs), and facilitating market linkages for recyclable materials. HKS, another stakeholder which constitute trained team of women entrepreneurs from the Kudumbashree (Kerala’s poverty eradication and empowerment programme) fold recruited to provide technical services and solutions on solid waste management projects. It is responsible for collection, transportation, storage, segregation processing, disposal, and management of waste in collaboration with the respective LSGIs. Another stakeholder is MGNREGS (Mahatma Gandhi National Rural Employment Guarantee Scheme) for infrastructure development mainly establishment of Mini-MCFs and MCFs through convergence. Ayyankali Urban Employment Guarantee Scheme (AUEGS) in urban areas for the same purpose. In the process, the Green Kerala Mission assumes the central role with techno-managerial support, public awareness initiatives, monitoring and evaluation, and interdepartmental coordination (Government of Kerala, 2018). This assemblage represents what Dean (2010) describes as “assemblage governance.” It includes a variety of agencies working collaboratively at multiple scales and directed by various logics of action, yet connected through an integrated purpose and complementary responsibilities. Operational activities are decentralized while strategic control is centralized. This approach is driven by subsidiarity and collaborative involvement.

### 4.5 The Green Kerala Mission: institutional context

The Green Kerala Mission, officially launched on December 8, 2016, constitutes a flagship initiative within Kerala’s sustainable development strategy, forming one of four core missions under the “New Kerala” vision for holistic and inclusive development. Guided by the motto “Water, Hygiene, and Harvest,” the mission articulates an integrated approach to environmental governance encompassing three sub-missions: (a) agriculture and organic farming, (b) sanitation and waste management, and (c) water conservation and security. This mission’s approach toward operational actions shall be considered under its embedded sense of socially integrating environmental actions toward mobilizing citizens as active participants within state initiatives toward sustainability, which aligns with recent trends toward environmental governance and deliberative democracy (Fischer, 2000), where state intervention can be empowered by citizens but not subject to market or technocratic solutions. The mission’s operational philosophy emphasizes socially embedded environmental action, seeking to mobilize citizens as active participants in state-led sustainability initiatives and the approach reflects broader trends in environmental governance toward participatory and deliberative democracy, wherein state capacity is augmented through citizen involvement rather than replaced by market mechanisms or technocratic interventions. Key programmatic interventions comprise the Green Law Understanding Program, which has significantly enhanced public awareness on environmental legislation and individual responsibilities. The mission employs contemporary technologies including digital monitoring platforms, GIS mapping, and mobile applications to facilitate transparency, accountability, and data-driven decision-making across governance levels. Importantly, the Green Kerala Mission presents environmental sustainability not as a techno-managerial problem amenable to purely technical solutions, but as a socio-political endeavor requiring behavioral transformation, institutional coordination, and cultural shifts in environmental consciousness.

### 4.6 Genesis and institutional positioning of Haritha Karma Sena/green task force

The Haritha Karma Sena was constituted in 2017 under orders from the Government of Kerala, through the Green Kerala Mission. The local self-government institutions were entrusted to form HKS units at each ward level for two purposes: to enable decentralized waste management and to provide sustainable livelihood opportunities to women from economically marginalized sections. The programme has emerged as one of the single largest women-led environmental services networks in the country today, with over 36,000 workers in Kerala’s local bodies. Recruitment into the HKS is through Kudumbashree, Kerala’s famous community-based women’s organization. Kudumbashree functions through three-tier units: the neighbourhood groups (NHGs) at the ward level, area development societies (ADSs) at the panchayat or municipal level, and community development societies (CDSs) at the district level. This ensures a deeply entrenched institutional base for the HKS. This linkage with established networks has been crucial in gaining legitimacy, ensuring operational efficiency, and acquiring social acceptance-all of which will be elaborated further in the section on analysis of social capital (Kudumbashree Mission, Local Self Government Department, Government of Kerala, n.d.).

### 4.7 Operational model and workforce structure

Haritha Karma Sena is based on a semi-formal door-to-door collection of solid wastes with well-defined roles and responsibilities to ensure efficiency and accountability. The two categories of workers are green technicians and green supervisors. The green technicians are the trained waste collectors deployed in all wards, usually two per ward, each serving at least 250 households a month. Their daily rounds comprise not just the collection itself but also imparting the necessary education to households for source segregation. The wastes after collection would be taken to Mini Material Collection Facilities, or Mini-MCFs, which serve as decentralized processing points. Oversight is performed by green supervisors, personnel with graduate qualifications who oversee operations across approximately fifteen wards. Their duties extend beyond monitoring collection activities to include administrative functions such as user fee collection, preparation of operational reports, quality assurance, and coordination with local self-government institutions (LSGIs) and representatives of the Clean Kerala Company (Local Self Government Department, Government of Kerala, 2021).

### 4.8 Training and capacity building

The study explores the operational effectiveness of HKS and credits its success to comprehensive training at EKSAT, or Empowerment through Knowledge, Skill, Attitudinal Change, and Traits. EKSAT is a residential training center that offers an intensive three-day course composed of topics targeted to multiple competency areas. Initial modules focus on the technical competencies of correct waste segregation, safe handling of various waste streams, following safety procedures, and the use of proper collection equipment. The next attention is given to health and safety: trainees learn about occupational hazards, personal protective equipment such as gloves and masks, ergonomic practices to avoid strain, and basic first-aid procedures. Another component involves communication skills. The program includes techniques for household engagement, conflict resolution, community mobilization, and instruction of the public. Personal development is the final component. Subjects include leadership, stress management, resource mobilization, and basics of entrepreneurship. Field excursions afford trainees experiential learning opportunities. Visits to well-managed waste systems, made in collaboration with local urban authorities, allow trainees to observe and analyze operational practice in context. The integrative cross-cutting design of the program corresponds to the assertion that waste management goes beyond the technical know-how. Effective performance depends on social competence, self-efficacy, and entrepreneurial mindset, all the more for personnel new to formal employment and stakeholder interaction. As expressed by Foucault (1991), the program can be characterized from a Foucauldian perspective as a “technology of the self,” shaping workers’ dispositions and capabilities in support of their conversion into new roles within environmental work.

### 4.9 Waste processing infrastructure and value chains

The HKS’s operational model integrates a hierarchical infrastructure of waste processing facilities including mini material collection facilities (Mini-MCFs) which is Ward-level temporary storage facilities for nonbiodegradable waste collected by HKS members. It reduces the physical burden on workers by establishing intermediate storage points, preventing the need for long-distance transportation after each collection round. Apart from mini-MCF they have material collection facility (MCFs), Panchayat or block-level hubs for storing, segregating, and consolidating dry waste from mini-MCFs. MCFs conduct primary segregation into recyclable categories (paper, plastic grades, glass, metals) and nonrecyclables. Recyclable materials are sold to Clean Kerala Company at predetermined rates and reviewed periodically on the basis of market conditions and for shredding the plastics there are resource recovery facilities (RRFs). RRF are district or regional-level mechanized centers equipped with shredding machines, baling machines, dust collectors, and conveyor belts for secondary segregation and processing of nonbiodegradable waste from MCFs. RRFs separate high-grade recyclables (sold to recycling industries), low-grade plastics (shredded for road construction applications), and nonrecyclables (sent to cement factories for coprocessing or safe disposal). This infrastructure embodies principles of the circular economy by maximizing resource recovery and minimizing landfill dependence (Clean Kerala Company Limited, n.d.).

### 4.10 Revenue model and economic sustainability

Along with waste collection and segregation, HKS members undertake various income-generating activities, such as recycling and producing value-added products from waste, manufacturing environmentally friendly materials, maintaining waste disposal mechanisms, organic farming, compost production, and renting environmentally friendly equipment. These activities not only diversify income sources for the members but also strengthen the program’s alignment with Kerala’s Mission Zero Waste and the United Nations Sustainable Development Goals (SDGs). The Haritha Karma Sena Micro Enterprises have various sources of income including user fees collected from households, user fees collected from institutions, the purchase of organic waste compost (for providing to farmers through farm projects at the LSG level), and revenue from the sale of nonbiodegradable waste and they also have the provision of starting their own green micro enterprises. In the financial year 2023–24, user fees and scrap sales generated approximately ₹22.43 crore showing financial viability at scale. This economic sustainability distinguishes HKS from grant-dependent or philanthropic waste management initiatives, enabling longer-term institutionalization and reducing vulnerability to funding fluctuations (Local Self Government Department, Government of Kerala, 2021). By facilitating income generation and skill development, HKS functions as a platform for enhancing women’s career trajectories within the semi-formal waste sector.

### 4.11 Digital governance infrastructure

KELTRON, the Kerala State Electronics Development Corporation and a government-operated technology enterprise, joined hands with the Green Kerala Mission, Suchitwa Mission, and KILA (Kerala Institute of Local Administration) to develop the Haritha Mithram application. This represents a major technological leap in the management of solid waste-a centralized digital hub connecting households, HKS workers, local self-government institutes (LSGIs), and state offices. The application provides the following features via its integrated modules:

- Household registration is conducted via QR codes, capturing location and occupancy data and waste estimates both at residential and institutional levels.
- Real-time tracking of collection routes, their schedules, and status for managerial and LSGI officials’ supervision.
- User Fees are processed online, reducing cash handling, minimizing leakages, and enabling transparent financial tracking.
- The GIS mapping demonstrates the location of MCFs, mini-MCFs, RRF plants, composting units, and biogas installations, thus helping do the exact amount of planning and creating logistics required.
- The grievance system allows citizens to report issues, request special collections, or provide feedback with strict timelines for responding by LSGIs.
- Dashboards visualize information on service coverage, waste composition, revenue, facility utilization, and compliance on a range from ward level to the Chief Minister’s office. (Local Self Government Department, Government of Kerala, n.d.).

The Haritha Mithram initiative is one such example of what is described as “digital governmentality” (Kitchin, 2014). In this regard, digital tools are not merely used for administrative purposes but also for influencing behavior and creating environmentally conscious citizens. Transparency and accountability flow from this arrangement. Public information on waste makes it easier for citizens to monitor. Employees and officials are held to performance standards. The system is two-way: citizens comply with regulations on segregation and fees, while HKS teams and LSGIs are accountable for any violations. The household data is tracked digitally, including the history of collection and the status of payments. The responsibility is now immediate and concrete, with families being made visible participants. Their eco-friendly behavior is now open to evaluation by the concerned authority. Data also supports informed decision-making for policy. Aggregated data metrics inform policy adjustments, resource reallocation, and bottleneck correction. For instance, waste mapping results highlight infrastructure requirements or behavior change campaign targets.

Citizen surveillance is operationalized via grievance systems that make individuals tools of state surveillance. Citizen reporting mechanisms can be interpreted as institutional strategies intended to foster a collective sense of responsibility. However, contradictions arise. The ideal of worker autonomy can conflict with pervasive surveillance, since routes, activities, and performance data are tracked. Does this discipline cross a threshold? This should be explored in future research. Do these technologies empower workers via a sense of professional identity, or do they further reinforce managerial control? This is at the very core of the digital labor debate (Moore and Robinson, 2016).

### 4.12 Participatory waste governance and civic engagement

To achieve effective waste management, there is a need for continued collective action on the part of different categories of stakeholders, such as households, commercial organizations, educational institutions, residents’ associations, and government officials. The Kerala approach to decentralization recognizes that technical systems by themselves have limited use without changes in behavior and community ownership (Government of India, 2016). The “Garbage-Free New Kerala: People’s Campaign” (Malinyamuktham Navakeralam), launched from October 2, 2024, to March 30, 2025, is one such example of Kerala’s dedication to mass mobilization in the pursuit of environmental goals. The campaign brought together actors at different levels of governance from district collectors to Grama Panchayat presidents to leaders of neighborhood groups in a collective assessment of the existing waste management systems, identification of gaps, and implementation of model projects. This inter-departmental approach is an acknowledgment of the fact that effective waste management requires mass participation that goes beyond the government bureaucracy to include educational institutions, non-governmental organizations, residents’ associations, self-help groups, and media organizations (Government of Kerala, n.d.).

The decentralized waste management system in Kerala is based on a primary policy of source separation by every family. Waste is classified into biodegradable, nonbiodegradable, and hazardous materials. Biodegradable waste is handled at the household level using techniques such as aerobins, pipe composting, pit composting, or biogas plants. The waste management service (HKS) collects only cleaned and separated nonbiodegradable waste. The framework is oriented toward promoting ecological citizenship and increasing environmental awareness.

Mini material collection facilities (Mini-MCFs) at the ward level bring forth a new paradigm in waste management at the local level. Mini-MCFs reduce the burden of HKS collectors and provide concrete, observable points of waste management in the community. The success of Mini-MCFs depends on educational objectives; otherwise, problems like dumping of mixed waste, misuse, or lack of maintenance could occur. The best possible performance of Mini-MCFs can be achieved through shared governance, which involves cooperation between local authorities, neighborhood associations, schools, resident associations, and volunteers. This is an example of polycentric governance (Ostrom, 1990).

### 4.13 Information, Education, and Communication (IEC) strategies

The need for proper waste management practices requires continuous education through various mediums. The staff of HKS are primarily responsible for environmental education. They visit homes, conduct school-based programs, and hold community workshops. The areas of training include waste reduction, segregation, composting practices, and recycling basics. Peer education will also be effective, as these staff members are local and come across as more approachable than experts (Government of Kerala, 2022). Research in participatory development and environmental governance has perennially revealed that the presence of women enhances community adherence, collective responsibility, and sustainability outcomes due to their higher levels of social connectedness and informal influence within households. Thus, the central role of women in teaching and learning about waste is not due to the essentialist assumption, but rather due to gendered social relations, which place women as central actors in mediating between policy guidelines and daily household activities.

Additional Information, Education, and Communication (IEC) activities support these efforts:

School programs: waste management is incorporated into the curriculum, with schools setting up composting sites and students engaging in clean-up campaigns.

Media campaigns: television, radio, print media, and social media communication campaigns focus on individual and collective responsibility in waste management.

Community activities: organized waste-free festivals, exhibitions of products made from recycled materials, and recognition of high-performing households and neighborhoods.

### 4.14 Structuration and institutional transformation

The 2018 solid waste management policy in Kerala clearly articulates the key objectives of HKS. The policy promotes decentralized solid waste management and, at the same time, aims to provide economic empowerment for women. However, HKS has brought in key hidden consequences, called latent functions, not articulated in its objectives but are highly significant in the larger societal context, as highlighted by Merton (2016). The following sections discuss the hidden aspects of HKS through the prisms of structuration theory, governmentality, and social capital.

Drawing on Giddens (1984), the HKS can be interpreted as an institutional site wherein the recursive relationship between structure and agency is potentially reconfigured within Kerala’s decentralized waste governance framework. Structuration theory conceptualizes social structures as both the medium and outcome of social practices; accordingly, the policy architecture governing HKS establishes a set of rules, norms and resources allocation that simultaneously enable and constrain action. At the structural level, the Kerala Solid Waste Management Policy (2018) and associated operational guidelines formalize waste management through clearly defined roles, user-fee mechanism, and integration with local self-governments. These institutional arrangements signal a shift towards the bureaucratic organization of waste work, positioning it within the domain of regulated public service rather than informal labour. Such formalization can be read as creating the conditions for the reconstruction of waste work as socially legitimate and institutionally embedded.

From a structuration perspective, however, the significance of HKS lies not solely in its formal design but in the possibility of its enactment through practice. The policy framework anticipates routine interactions between HKS workers and household, the enforcement of waste segregation norms, and participation in localized governance processes. These repeated practices constitute the site through which structures are reproduced or potentially transformed. While empirical verification lies beyond the scope of this document-based analysis, the framework itself suggests that HKS workers are positioned as agents capable of mediating governance at the community level.

This dynamic assumes particular importance in relation to historically entrenched social hierarchies, including caste-based associations with waste work. Although the policy does not explicitly address caste, its emphasis on institutionalization, standardization, and integration within decentralized governance structure may be interpreted as creating opening for the gradual destabilization of stigma. In this sense, HKS can be conceptualized as a potential structural intervention, wherein the reorganization of labour within formal governance system rearticulate the social meaning attached to waste work.

### 4.15 Social capital formation

Putnam et al. (1994) defines social capital as “features of social organization such as networks, norms, and trust that facilitate coordination and cooperation for mutual benefit.” An analysis of Kerala Solid Waste Management Policy (2018) reveals that its emphasis on door-to-door waste collection through HKS creates fertile condition for the formation of social capital; bonding, bridging, and linking social capital. At the level of bonding social capital, HKS members get opportunity to develop strong intra-group solidarity through shared work experiences, collective problem solving and mutual dependence. More significantly, the programme facilitates the development of bridging social capital by positioning HKS workers in continuous interaction with households across diverse social and economic categories. Door to door waste collection is not a one time but a repetitive, relationship-based engagement. These interactions create the potential to transform initially hierarchical relationships into more reciprocal ones, thereby fostering trust and cooperation over time. Thus, Door-to-door visits cross old divides, creating weak ties (Granovetter, 1973). Then compliance will be achieved not only through regulation but through social relationships and moral persuasion. HKS have the potential that proves state programs can grow social capital, not just use what’s already there. It starts with Kudumbashree’s bonding ties but creates fresh bridging and linking capital through daily operations. This fits research that rejects simple state-vs-society splits, showing how government can spark, shape, and strengthen social networks (Evans, 1996; Uphoff, 2000).

At a broader level, the policy enables linking social capital by formally connecting HKS workers with institutional actors such as panchayat, municipalities, corporation, health inspectors and Kudumbashree officials. This vertical integration provides workers with a channel to access resources, information, and a degree of institutional recognition that would otherwise be unavailable in informal waste work. The uniform, ID cards, and formalized roles further strengthen this linkage by symbolically and practically embedding workers within governance frameworks.

However, an analytical reading also suggest that the formation of social capital may be uneven and contingent upon contextual factors. While the policy creates structural opportunities, the actual realization of social capital depends on factors such as community participation, local governance efficiency, and the persistence of caste- and occupation-based stigma. In context where residents resist cooperation or continue to view waste workers through entrenched social hierarchies, bridging social capital may remain weak, limiting the transformative potential of the program.

### 4.16 Governmentality and environmental subjectivity formation

Foucault’s concept of governmentality, developed in 1991, provides us with a very effective perspective on how Haritha Karma Sena becomes a part of environmental regulation. “Governmentality” is concerned with “conduct of conduct”-methods, techniques, and rationales that regulate how individuals and how social groups conduct themselves. More significantly, Foucault moves attention away from the policing and prohibition conception of power and emphasizes the productive aspect of power whereby new knowledges, new ways of thinking about ourselves, and new social practices emerge. Agrawal’s (2005) approach of environmentality is an extension of governmentality in which it examines how individuals become environmental subjects, bearing responsibility not only on others but also on themselves. This is not necessarily an experience triggered by force, punishment, or compulsion. Rather, it is a process in which institutions, schooling, and daily routines make it seem natural, necessary, and even enticing. In other words, it is in the nature of institutions, schooling, and daily performance of actions in which individuals experience a sense of being induced to care for nature, which is far less coercive than punishment.

The HKS exemplifies multiple dimensions of environmental governmentality:

Decentralization as dispersed governance: the meaning Foucault gives here is that power is not concentrated in one big machine-like structure. It moves through many hands and at many locations. This is also the case in HKS, where the management of waste is not consolidated in one department but in several stakeholders; Kudumbashree units, the Clean Kerala Company, the Suchitwa Mission, and household units etc. The state does not directly control the garbage but controls a network of actors, every one playing its part within the larger system. This is how the state is able to govern from a distance, where the central state is able to control people’s behavior not through direct commands but through the design of policies and frameworks at the institutional level (Rose and Miller, 2013).

Technologies of government: foucauldian governmentality emphasizes the use of technical instruments, including standards, audits, budget, inspection and performance measurement, by which the governance is operationalized. In decentralized waste governance, HKS uses a number of these technologies. The system of user fees reorganizes waste management as a collective fiscal charge, and incorporates households into the economic rationality of environmental services. The Haritha Mithram application of digital monitoring is the means of increasing visibility, traceability, and accountability of performance. Household registration based on QR-codes makes populations administratively legible and thus allows systematic control. The training programmes also entrench procedural compliance because they impart standardized operational knowledge to the workers. These tools are used to control behavior in an indirect manner by arranging routines, responsibilities, and quantifiable results instead of coercing directly.

Self-regulation: advanced liberal rule operates by producing self-governing subjects who willingly coordinate their actions in alignment with policy objectives. Within this framework, HKS seeks to promote behavior change by integrating practices such as waste segregation, composting, and environmental hygiene into everyday domestic routines. Rather than relying on coercive or repressive measures, conformity emerges through processes of normalization, whereby environmentally responsible behavior is encouraged as a routine aspect of citizenship (Rose, 1999). Ecofriendly oriented campaigns like the “Garbage free Kerala” make the environmental purity an icon of civic responsibility and shared pride, thus making citizens feel themselves participating in the ecological governance.

Normative formation and identity: through daily activities, environmental subjectivities are shaped and molded into habits and identities. Source separation, composting, contact with HKS staff, payment of fees, and cleaning campaigns inscribe environmental responsibility into the individual’s identity. Individuals are positioned as environmental subjects within the governance framework. The shift from seeing garbage as a personal problem to a community responsibility, and from individual accountability to ecological subjectivity, encapsulates the process of ecological identity formation.

Knowledge production and expertise: governmentality is characterized by knowledge regimes that specialize in problem definition and solution prescription. HKS relies on diverse expertise: scientific knowledge about the composition and processing of waste; pedagogical methods aimed at inducing behavioral change; managerial skills in service delivery and performance; and local knowledge about neighborhood dynamics and household practices.

Decentralized waste governance can be examined by the concept of governmentality developed by Michel Foucault because it stresses the existence of power in everyday practices, behavioral norms, and self-regulation, instead of being concentrated on coercion through the state power. In solid waste management, the governance is implemented by mechanisms like door-to-door collection, requirements of source segregation, grievances redressal system, and so forth, which depict how governance is practiced between citizens and frontline workers as mediators of regulatory rationalities. But governmentality studies can no longer be left as descriptive accounts of the circulation of power. Rather they need to critically address the political implications of the governance arrangements, which include the issues of inequality, agency and contestation (O'Malley et al., 1997). Applied to this study, this perspective can be interpreted as creating conditions that may both enable forms of empowerment and position workers as intermediaries within governance processes. However, assessing the extent to which these dynamics are realized in practice requires empirical research among HKS workers. In this way, the framework goes beyond the consideration of governance as either entirely emancipatory or entirely disciplinary, and places governance in a dynamic arena of negotiation, responsibility and power.

### 4.17 The caste-embedded waste economy in India

India’s waste economy remains structurally organized by caste hierarchies that relegate stigmatized waste handling labor onto Dalit communities, and especially Dalit women. Despite legal prohibition, manual scavenging-manually removing human excreta from dry latrines-continues to employ approximately 1.3 million people, and largely Dalit women, in this dehumanizing labor. More broadly, municipal waste collection, sweeping, and disposal labors are disproportionately delegated to Dalits who work under precarious conditions; hazards are common, compensation is poor, and social stigma is widespread (Gill, 2009; Doron and Jeffrey, 2018). Harriss-White (2020) analyzes the waste management system of one small town in order to show how caste, class, and patriarchy together shape India’s waste economy. This study shows that Dalit and Adivasi women hold the lowest and most hazardous positions in the waste hierarchies, accomplishing manual tasks without formal contracts, adequate facilities, or formal recognition, and face risks related to illness, harassment, and violence. The state’s fragmented bureaucracy, weak institutional coordination, and chronic underinvestment sustain the reliance on informal labor and at the same time reproduce structural inequalities. Due to this fact, waste work is not a temporary occupation instead, it is socially bounded, gendered, and exclusionary concerning citizenship and dignity.

This structural embedding of trash work in caste hierarchies reflects broader patterns wherein purity and pollution distinction structure social relations and labor divisions in India (Saldanha et al., 2022). Bodily waste, dead animals, and the avoidance of pollution are relegated to those considered ritually impure, thus naturalizing their subaltern social position through a rationale of religion. Economic liberalization has not abolished these hierarchies but has often rearranged them, with waste labor continuing to be largely performed by Dalits, while new forms of sanitation labor create precarious possibilities for social mobility that are still circumscribed by caste (Mahalingam and Selvaraj, 2022).

### 4.18 Empirical imperatives for caste analysis

While the HKS framework does not explicitly engage with caste, its institutional design opens up the possibility for reconfiguring caste-linked stigma associated with waste work. Recruitment through Kudumbashree potentially enables broader social representation, bringing women from diverse caste backgrounds into a formally organized system. This institutionalization, supported by uniforms, remuneration, and integration into local governance structures, may contribute to repositioning waste work from a caste-ascribed occupation to a recognized public service. However, such shifts should not be assumed to translate into substantive transformation. The apparent inclusivity of the framework may coexist with underlying continuities, particularly if marginalized caste groups remain disproportionately represented in such roles. In this sense, HKS can be understood as creating conditions for the possible renegotiation of caste relations, while also leaving open the question of whether these structural interventions are sufficient to disrupt deeply embedded social hierarchies. These questions require ethnographic methods, in-depth interviews, and longitudinal observation extending beyond this paper’s documentary scope. The analysis offered here establishes HKS’s formal mechanisms for professionalizing and dignifying waste work while acknowledging that document-based research cannot access lived experiences, cultural meanings, or subtle dynamics of social recognition and exclusion.

### 4.19 Haritha Karma Sena as a structural intervention

HKS is portrayed as a state-led effort that seeks to challenge prevailing behavioral trends. This is achieved through official employment terms and professional development opportunities. In the waste management industry, gender imbalances, social norms, and structural inequalities influence the allocation of employment opportunities between women and men. Women are mainly responsible for disposing of domestic waste. However, men have always dominated the organized waste management sector and are also at the forefront of decision-making (Mwangi et al., 2021; Amoah et al., 2023). Despite women making up almost half the population in a given environment, their contribution to waste management remains limited to informal sectors, which are often unpaid or minimally paid. On the other hand, men hold the better-paid administrative and supervisory roles in the sector. This is observed in South Asian environments, where women have long been responsible for managing domestic waste and street cleaning activities. But as soon as these roles are recognized or monetized, men take over from women, and women are left with the less-paid and less-secure jobs (Dankelman et al., 2009; Dos Muchangos and Vaughter, 2019). The Kerala HKS is a significant exception to the general patterns of the region. In contrast to the typical model, HKS does not offer the usual type of salaried employment. Rather, employees function as self-employed entrepreneurs in Kudumbashree micro-enterprises. However, this system also brings a significant degree of formalization and professionalization to what was previously a form of informal waste labor. Women are still at the center and not on the periphery as waste management becomes institutionalized. They are at the center as important actors, who are recognized, trained, and supported. HKS brings about a transition from hidden, insecure informal work to visible, supported entrepreneurial work.

The HKS framework repositions waste-handling activities as skilled environmental entrepreneurship that requires technical proficiency, social engagement competencies, and business management skills, rather than as stigmatized labor traditionally associated with caste hierarchies or performed by women out of economic necessity. And also, HKS differs from the informal waste-picking activities that lack any kind of labor security and are not supported by any institutional framework and, on the other hand, the formal municipal employment that involves formal jobs with salaries and benefits. Instead, it proposes a hybrid model of formalization that involves self-employment recognized and supported by institutional frameworks. This approach maintains the flexibility of self-employment while providing stable training, equipment, market access, and protection. In doing so, HKS places women at the forefront of its activities and thus directly opposes the common South Asian trend of gender displacement in the process of formalization.

HKS mainly recruits from Kudumbashree networks and not from already existing municipal waste workers or pickers. This approach fosters an emergent labor force that lacks any prior caste association with waste work. Kudumbashree includes women of all castes; however, the focus of the program on economic empowerment and self-sufficiency recontextualizes waste work as a means to autonomy and not as a caste duty. As the concept of empowerment is necessarily multidimensional, there is a danger of overlooking this transformative potential of the multidimensional waste management programs centered on women by focusing the analysis exclusively on economic indicators. Although income generation and livelihood security are the important elements, the dimensions of empowerment include social recognition, power to make decisions, collective agency, acquisition of skills and change in gendered power relations. The analysis of these overlapping dimensions allows for a more nuanced understanding of how involvement in decentralized waste governance has the potential to contribute to shifts in women’s identities, enhance their visibility, and create conditions that may support greater bargaining power within households and communities.

This would take women engagement out of the limited income-based paradigm but provide them with a broader process of structural and relational transformation and in the process identify the more emancipatory opportunities contained within models of community based environmental governance. Future studies can address this aspect and contribute to the literature on women empowerment.

State recognition and public visibility: HKS employees are recognized as official representatives of local self-government bodies. Their uniforms and identity cards formally identify them as government officials and service deliverers, rather than marginalized workers. Public awareness campaigns actively recognize and celebrate their role in environmental sustainability. Public visibility can be interpreted as a mechanism through which shifts in the perception of waste workers from stigmatized outcastes to recognized environmental professionals and change agents may occur.

Economic viability: unlike the informal waste sector, which is often linked to unprofitable pricing, wage theft, and economic insecurity, HKS provides economic viability. Employees are assured of income through user fees, fixed rates for buying back recyclables, and the potential for creating value-added products from waste materials. The system also acts as a viable solution for the increasing burden of low- and middle-income countries regarding municipal expenditure on solid waste management. At the global level, it has been observed that in low- and middle-income countries, urban local bodies allocate 10–20% of the entire municipal expenses for the management of municipal solid waste. Out of these expenses, the cost for the collection and transportation of waste contributes to about 60–80% of the entire expenses (Guerrero et al., 2013; Kaza et al., 2018). In response to these structural constraints HKS reconfigure the economic burden by redistributing responsibilities across local governments, households and various related stakeholders. In this sense, HKS operates as a form of fiscal and governmental rationality and become an economically feasible solution.

Institutional embeddedness: HKS has institutional ties with various stakeholders. This strong institutional embedding provides workers with organizational support systems, grievance redressal mechanisms, and increased political visibility, which are not available to informal waste pickers.

HKS is a significant departure from the usual strategies employed in the Indian urban setting to formalize the informal sector of waste pickers. Cities such as Pune, Bengaluru, and others have sought to recognize the value of the waste pickers through approaches such as cooperatives, identity cards, and direct engagement with the waste management systems of the city (Chikarmane and Narayan, 2005). However, these integration activities face significant challenges. Middle-class community members are often skeptical of the conventional pickers and display hesitation to interact with them. Municipal contractors are resistant due to political reasons. Caste discrimination limits the mobility and market access of the pickers. The promised benefits of institutionalization, such as health insurance or proper pricing, are not achieved. In addition, the rules-in-form do not always align with the rules-in-use (Pastor et al., 2024). The Haritha Karma Sena (HKS) adopts a distinct approach to waste management governance in the Kerala context, where the presence of a large, organized informal waste worker sector is relatively limited. Rather than focusing on the integration of informal labour into formal systems, HKS establishes a new cadre of trained women workers within an institutional framework. This approach leverages the existing social infrastructure of Kudumbashree, enabling the organized deployment of labour through decentralized governance structures. By building such an institutional arrangement from within the state-supported community network, HKS achieves a level of coordination and statewide reach that is often difficult to attain in models that rely on the formalization of pre-existing informal sectors.

### 4.20 Multidimensional SDG integration of Haritha Karma Sena

The United Nations’ Sustainable Development Goals (SDGs) point out the importance of sustainable waste management as a key driver in achieving a balanced approach to sustainability (United Nations, 2015). Waste management, when done correctly, touches upon environmental, economic, and social dimensions of sustainability. At the core of sustainable waste management is the need for systems that are environmentally effective, economically feasible, and socially acceptable. Environmentally, the goal is to reduce harm to the planet (Elsheekh et al., 2021).

An analysis of Kerala’s decentralized waste management model through Haritha Karma Sena (HKS) indicates its broad alignment with several SDGs, particularly those related to gender equality, decent work, sustainable cities, and responsible consumption.

The programme can be linked with SDG 5 (Gender Equality) and SDG 8 (Decent Work and Economic Growth) as the HKS framework emphasizes women’s participation in decentralized waste management activities and livelihood generation. The institutionalization of waste collection and segregation through HKS reflects attempts to create organized livelihood opportunities and enhance women’s participation in public and environmental governance spaces.

Similarly, the decentralized model of waste collection, segregation, composting, and recycling corresponds with the objectives of SDG 11 (Sustainable Cities and Communities) and SDG 12 (Responsible Consumption and Production). The emphasis on source-level waste management, material recovery, and decentralized processing in Kerala’s waste management framework reflects broader sustainable development principles promoted within the SDGs. References to biogas plants, organic waste treatment, and reduction of landfill dependency within policy frameworks may also be understood in relation to SDG 13 (Climate Action) and SDG 7 (Affordable and Clean Energy).

The establishment of decentralized waste processing facilities, material collection centres, and digital platforms such as Haritha Mithram further indicates the infrastructural and governance dimensions of Kerala’s decentralized waste management approach. The programme framework also stresses collaboration among local self-government institutions, Kudumbashree, residents’ associations, schools, and recycling agencies, reflecting participatory approaches to local governance.

Thus, the alignment between HKS and the SDGs is understood here not as an empirical measurement of development outcomes, but as an analytical interpretation based on policy objectives, programme design, and institutional frameworks associated with Kerala’s decentralized waste management model.

### 4.21 Scalability and contextual specificity

The contextual preconditions raise important questions about HKS’s replicability in contexts lacking similar political, social, and institutional foundations. States with weaker local governments, lower social capital, more rigid caste hierarchies, or patriarchal norms restricting women’s public roles may find HKS’s model difficult to implement despite its technical design merits. Scalability research should identify which elements prove universally transferable versus which require contextual adaptation or substantial institutional development before implementation.

## 5 Conclusion

This paper examines Kerala’s decentralized solid waste management model through the institutional framework of Haritha Karma Sena (HKS), demonstrating how policy design reconfigures waste governance beyond technical service delivery. The analysis shows that HKS is constructed within policy discourse as a mechanism to embed environmental accountability at the household level while simultaneously formalizing and revaluing historically stigmatized waste labour. Rather than treating these as empirically established outcomes, the study interprets them as institutional intentions articulated through policy frameworks and operational guidelines. At the same time, a critical reading of these documents indicates unresolved tensions, including the conditions of semi-formal labour, the persistence of caste-linked stigma, and the implications of digital monitoring for worker autonomy and control. These contradictions highlight the gap between policy design and lived realities, underscoring the limits of document-based analysis. The significance of the HKS model, therefore, lies in its potential as a governance framework that reorganizes environmental responsibility and labour relations, while also pointing to the need for empirically grounded research to assess how these policy logics are negotiated, contested, and realized in practice.

### 5.1 Future research directions

The analysis of the Haritha Karma Sena (HKS) initiative provides a valuable policy blueprint and theoretical insight, but its long-term impact requires empirical validation. Future studies could investigate how the professionalization of waste work influences public and worker self-perceptions, particularly in relation to caste detachment, stigma reduction, and gendered recognition. Longitudinal research using Structuration theory would be especially useful for examining how the routinized practices of HKS members recursively reshape social norms and material structures surrounding waste labour. Another important avenue concerns the scalability of the HKS model: given its reliance on the Kudumbashree network’s social capital, comparative studies should assess whether similar outcomes can be achieved in regions lacking strong self-help group infrastructures or decentralized governance traditions. Finally, future inquiry should critically examine the dynamics of community participation and digital governance, focusing on how technologies such as the Haritha Mitram app influence worker autonomy, disciplinary pressures, and citizen compliance. Such research would help clarify whether decentralized waste governance can sustain inclusivity and responsiveness without coercion, thereby extending the theoretical and policy relevance of the HKS framework.
